# Supplementary material for: HAYSTAC: A Bayesian framework for robust and rapid species identification in high-throughput sequencing data
Source: PLoS Comput Biol. 2022 Sep 30;18(9):e1010493. doi: 10.1371/journal.pcbi.1010493 (PMC9555677; doi:10.1371/journal.pcbi.1010493)
Supplement: S1 Appendix — (PDF) [file pcbi.1010493.s001.pdf]

HAYSTAC: Supplementary Information

Evangelos A. Dimopoulos[1,*,✉], **Alberto Carmagnini**[2,*], **Irina M. Velsko**[3], **Christina Warinner**[3,4], **Greger Larson**[1], **Laurent A. F. Frantz**[2,5,#,✉], **and Evan K. Irving-Pease**[1,6,#,✉]

[1]The Palaeogenomics and Bio-archaeology Research Network, Research Laboratory for Archaeology and History of Art, University of Oxford, Oxford, UK.
[2]School of Biological and Chemical Sciences, Queen Mary University of London, London, UK.
[3]Department of Archaeogenetics, Max Planck Institute for the Science of Human History, Jena, Germany.
[4]Department of Anthropology, Harvard University, Cambridge, USA.
[5]Palaeogenomics Group, Department of Veterinary Sciences, Ludwig Maximilian University, Munich, Germany.
[6]Lundbeck Foundation GeoGenetics Centre, GLOBE Institute, University of Copenhagen, Copenhagen, Denmark
[*]Contributed equally
[#]Co-supervised this work

Supplemental Appendix. Calculation of false positive, false negative and true positive rates

For the calculation of the false positive, false negative and true positive rates we used the following formulas:

$$FP_r = \frac{FP}{FP+TN} \tag{1}$$

$$FN_r = \frac{FN}{FN+TP} \tag{2}$$

$$TP_r = \frac{TP}{TP+FP} \tag{3}$$

where: $TP =$ number of true positives, $FP =$ number of false positives, $TN =$ number of true negatives, and $FN =$ number of false negatives. For a true positive identification we require an abundance equal or higher than 0.01%.
