# Supplementary material for: HAYSTAC: A Bayesian framework for robust and rapid species identification in high-throughput sequencing data
Source: PLoS Comput Biol. 2022 Sep 30;18(9):e1010493. doi: 10.1371/journal.pcbi.1010493 (PMC9555677; doi:10.1371/journal.pcbi.1010493)
Supplement: S1 Fig — Memory remains constant as sample size increases, and runtime in most methods (other than Kraken2/Bracken and KrakenUniq) scales with the database size rather than input sample size). (PDF) [file pcbi.1010493.s002.pdf]

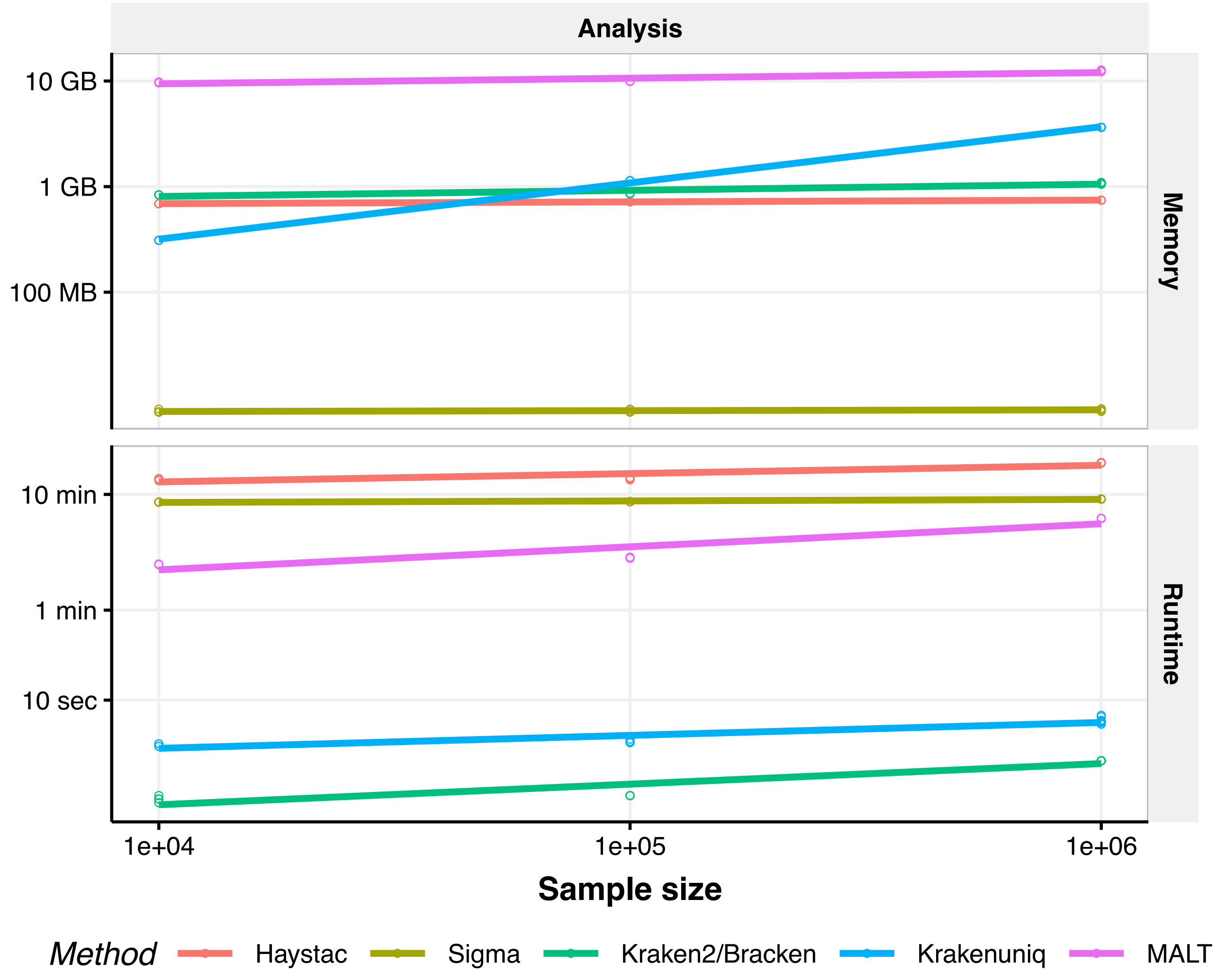

Supplemental Figure 1. Benchmarking for elapsed runtime and memory for HAYSTAC, Sigma, Kraken2/Bracken, Krakenuniq and MALT when analysing samples of 10 K, 100 K and 1 M reads against a database of 500 species. Memory remains constant as sample size increases, and runtime in most methods (other than Kraken2/Bracken and Krakenuniq) scales with the database size rather than input sample size).
