# Supplementary material for: HAYSTAC: A Bayesian framework for robust and rapid species identification in high-throughput sequencing data
Source: PLoS Comput Biol. 2022 Sep 30;18(9):e1010493. doi: 10.1371/journal.pcbi.1010493 (PMC9555677; doi:10.1371/journal.pcbi.1010493)
Supplement: S2 Fig — (PDF) [file pcbi.1010493.s003.pdf]

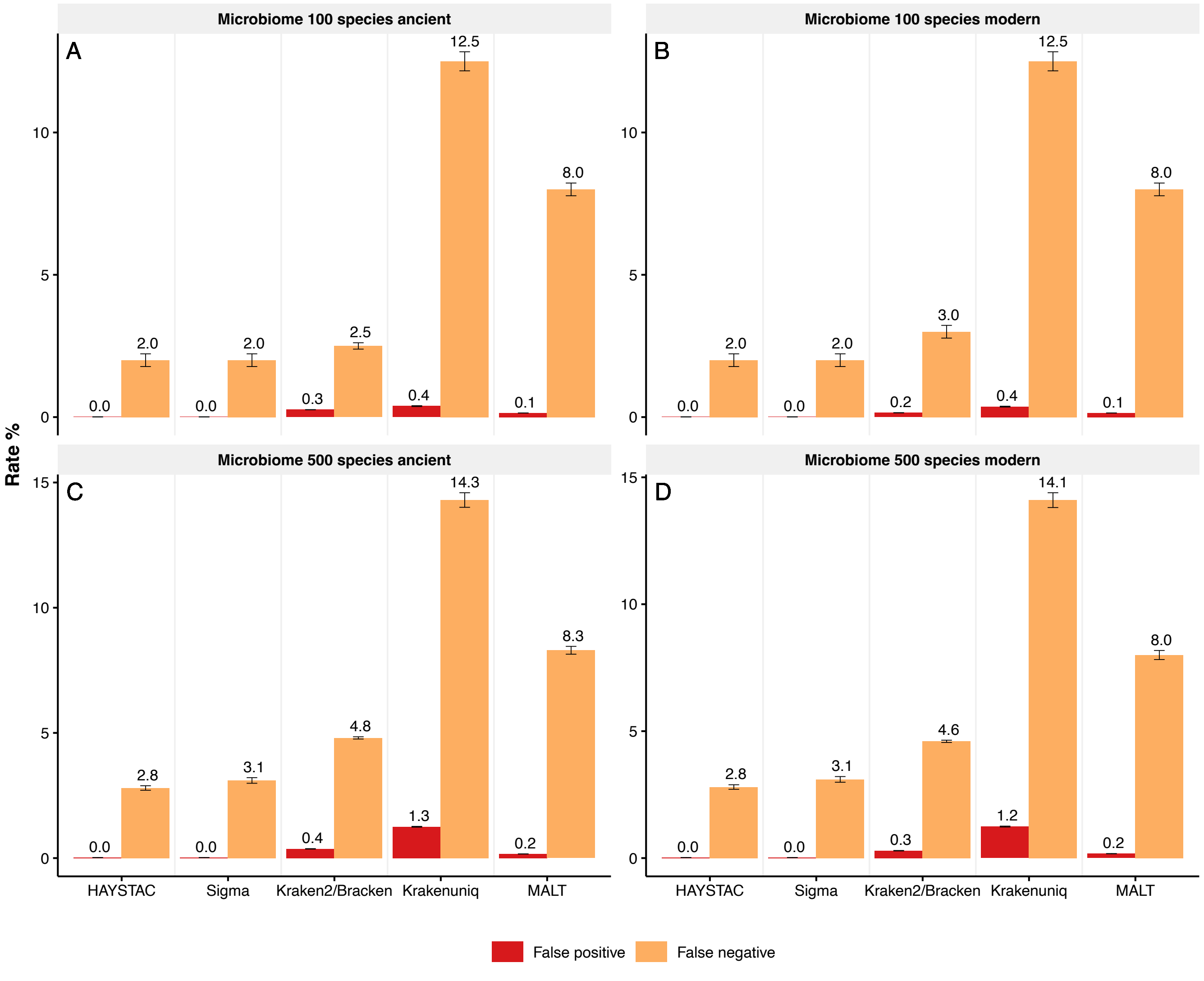

Supplemental Figure 2. False positive and negative rates per method for the simulated samples of the General Microbiome dataset.
