# Supplementary material for: HAYSTAC: A Bayesian framework for robust and rapid species identification in high-throughput sequencing data
Source: PLoS Comput Biol. 2022 Sep 30;18(9):e1010493. doi: 10.1371/journal.pcbi.1010493 (PMC9555677; doi:10.1371/journal.pcbi.1010493)
Supplement: S3 Fig — Mean count of false positive (red), false negative (orange), and true detected species (blue) in the simulated General Microbiome dataset of 100 species ancient (n = 2) (A), 100 species modern (n = 2) (B), and 500 species ancient (n = 2) (C) and 500 species modern (n = 2) (D). The dotted line represents the average number of simulated species in each set of samples, and the numbers above the error bars represent the mean species count. (PDF) [file pcbi.1010493.s004.pdf]

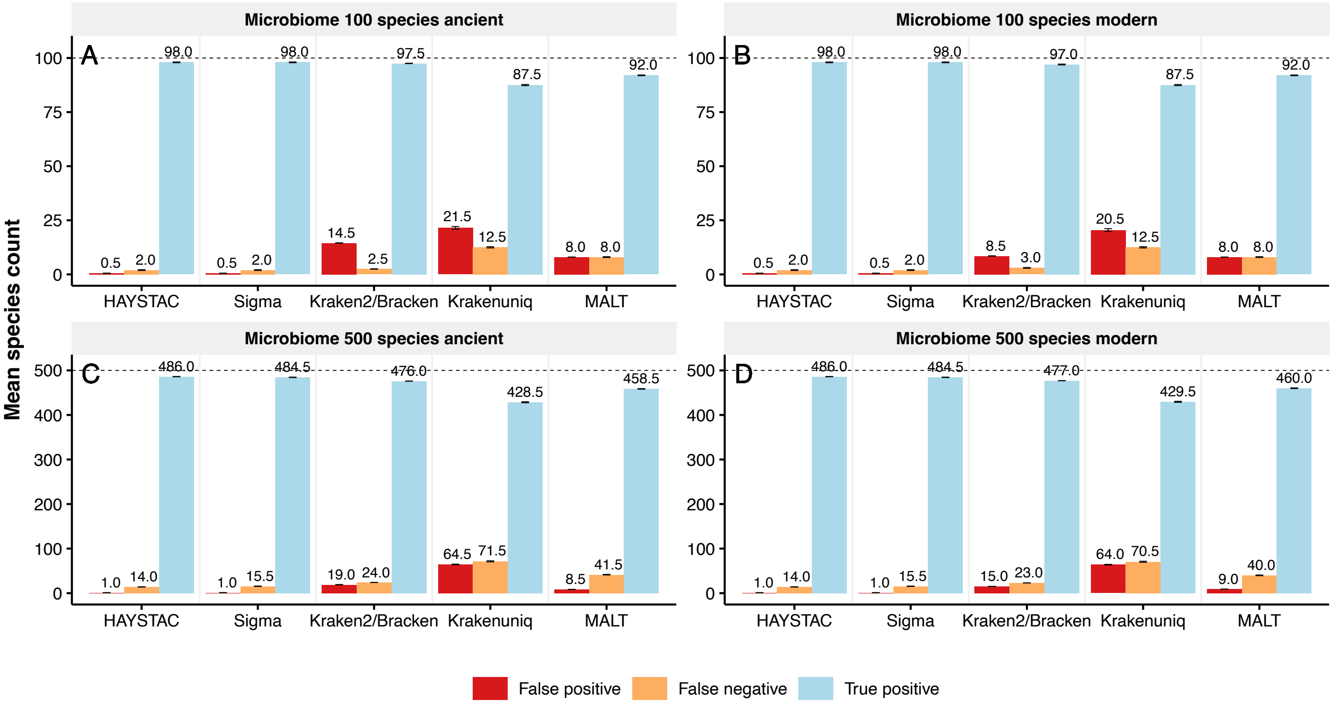

Supplemental Figure 3. Mean count of false positive (red), false negative (orange), and true detected species (blue) in the simulated General Microbiome dataset of 100 species ancient (n=2) (A), 100 species modern (n=2) (B), and 500 species ancient (n=2) (C) and 500 species modern (n=2) (D). The dotted line represents the average number of simulated species in each set of samples, and the numbers above the error bars represent the mean species count.
