# Supplementary material for: HAYSTAC: A Bayesian framework for robust and rapid species identification in high-throughput sequencing data
Source: PLoS Comput Biol. 2022 Sep 30;18(9):e1010493. doi: 10.1371/journal.pcbi.1010493 (PMC9555677; doi:10.1371/journal.pcbi.1010493)
Supplement: Attachment — Submitted filename: HAYSTAC_response_to_reviewers.docx [file pcbi.1010493.s019.docx]

Dear Niranjan and William,

Thank you for sending our manuscript for review, and for the helpful and constructive comments from the two reviewers. Below we list our responses to the reviewers’ comments.

**Reviewer #1**

**In lines 145 - 160 the authors write that HAYSTAC runs in "a single step".**

We have removed the “single-step” description of HAYSTAC as suggested by the reviewer (lines 149-150 of the clean PDF manuscript).

**HAYSTAC is not a single program, but rather a Python script that uses Snakemake to call eight different programs. This is a brittle construct.**

**While the use of bioconda and Snakemake ensure that all necessary versions of the programs are put in place when the tool is used, my worry would be that the HAYSTAC is "frozen in time" and moving forward it will be difficult to benefit from updates to any of the eight ingredient programs.**

**In contrast, the Kraken2/Bracken pipeline consists of two programs, and the described MALT/HOPS pipeline consists of two self-contained Java programs. In both cases, this is a much less brittle architecture.**

**I think that you should be forthright about the fact that HAYSTAC has many more moving parts than the other tools and discuss whether this is problematic or not.**

During the development of HAYSTAC we took efforts to balance the conflicting priorities of (a) minimising the number of external dependencies; and (b) maximising the number of analysis steps our software can automate. To offset the cost of having multiple dependencies, we ensured that all packages were installable via well maintained conda channels, to allow installation of HAYSTAC and all its dependencies with a single command. There are many bioinformatic software tools that are hard to install, and we have taken care to ensure that HAYSTAC is straightforward to setup.

However, we accept that pipelines which integrate multiple software dependencies often have high coupling between modules, which can result in a brittle architecture if the underlying dependencies change. To mitigate this, we have pinned exact software versions for all of our dependencies in the bioconda recipe for HAYSTAC, which ensures that only fully compatible software versions are installed.

The reviewer is correct that this can result in dependencies that are "frozen in time"; however, we consider this architecture to be advantageous, as it allows HAYSTAC to benefit from software that is well tested and actively maintained by the open-source community. When new versions of our software dependencies become available, we can rapidly incorporate their improved functionality by running our integration tests, to confirm compatibility with HAYSTAC, and then update our bioconda recipe—which we have done on several occasions (the current release of HAYSTAC is the sixth).

We also note that HAYSTAC is not the only tool with embedded software dependencies. SIGMA is a wrapper around bowtie2 and Ipopt. MALT is a wrapper around MEGAN, which is itself a wrapper around blast. Both Kraken2 and Krakenuniq have wrapper scripts around several bash modules, especially for their database construction steps which request data from NCBI.

We added some additional text discussing the benefits of HAYSTAC’s computational infrastructure in lines 545 to 547 (of the clean manuscript PDF) in the first paragraph of the Discussion section, as well as in lines 794 to 800 in the “Computational architecture” subsection of the methods section.

**Reviewer #2**

**What is the rationale for choosing Kraken2/Bracken instead of KrakenUniq?**

We chose to use the Kraken2/Bracken pipeline because we were specifically interested in testing Bracken’s performance as a species identification tool. We thank the reviewer for their suggestion of also testing KrakenUniq and have added it to the set of software comparisons made in the paper. Regarding its computational performance, Krakenuniq required the highest amount of memory and was the slowest performing program for database construction, but it was the second fastest program (after Kraken2/Bracken) in the sample analysis test. As a metagenomic profiler, it produced fewer false positive identifications than Kraken2/Bracken and MALT, but it was outperformed by both HAYSTAC and SIGMA, both in terms of false positive and true positive identifications. We have added these results to the manuscript (lines 224-226, 277-281, 287, 312, 320, 344-345, 353-354, 388, 393 in the Results section of the clean PDF manuscript).

**The author claims to have developed a "rapid species identification" tool. According to my understanding, lines 516 to 520 support this claim. This relates to the use of reduced reference databases for genera, which reduces the computational time (350 times faster). As shown in figure 2, Kraken2 provides a faster run time for analysis. Including KrakenUniq in Figure 5 ("Genus specific database analysis") would enhance your claims, especially in reporting the computational time and accuracy over KrakenUnique in this case.**

We have added KrakenUniq to the analyses shown in both Figures 2 and 5. To further illustrate the computational performance of HAYSTAC when using a reduced reference database, we have added text to the manuscript containing benchmark results from running 1 million reads analysed against a database of the genus *Corynebacterium* (84 species in total). This highlights the dramatic speed improvement achieved when using a genus specific database, compared to the full 500 species database. We believe this is a fair comparison for HAYSTAC, as the performance results shown in Figure 5 indicate that Kraken2/Bracken, KrakenUniq and MALT have unacceptably high false positive rates when run with a reduced reference database.

**Please provide a brief explanation of why Sigma presented fewer false positives for modern DNA than Haystack in Table 2.**

This discrepancy in the performance between HAYSTAC and SIGMA is partly due to the use of a fixed abundance detection threshold (0.01% relative abundance). Among false positive species identified by HAYSTAC and not by SIGMA, most have abundances just above the threshold in HAYSTAC and just below the threshold in SIGMA. Using a higher threshold would change this result. We mention this response in the first paragraph of the Discussion section (lines 529 - 530 in the clean PDF manuscript).

When analysing modern data using default settings, HAYSTAC has a slightly higher rate of read misassignment than SIGMA, because SIGMA derives the number of maximum mismatches allowed for a valid alignment only from the base mismatch probability used by its probabilistic model (0.05). HAYSTAC calculates the maximum number of mismatches for a valid alignment based on both the base mismatch probability (0.05) and the average read length of reads in the library (e.g., 150 bp for the modern data, which allows for more mismatches). This is also the reason why HAYSTAC has fewer false positive identifications in ancient samples with shorter read lengths. The above response is also included in lines 525 to 537 (of the clean PDF manuscript) of the Discussion section.

This aspect of the performance of HAYSTAC can be easily optimised by reducing the base mismatch probability at run-time. However, for the purposes of our comparisons, we felt it most appropriate to compare all tools using their default settings, rather than fine tuning the results from our method to suit the test dataset.

**The code and data used to generate the figures in the paper are not provided. Where it is possible, such data/code should be made available in order to enhance reproducibility in the paper.**

Thank you for alerting us to this omission. The code used for all tests and figures presented in the manuscript are available here: <https://github.com/antonisdim/haystac_paper.git>

Best regards,
Evan K. Irving-Pease on behalf of the authors
